# Predictive Factors of Short-Term Related Musculoskeletal Pain in the Automotive Industry

**DOI:** 10.3390/ijerph182413062

**Published:** 2021-12-10

**Authors:** Ana Assunção, Vera Moniz-Pereira, Carlos Fujão, Sarah Bernardes, António P. Veloso, Filomena Carnide

**Affiliations:** 1 Biomechanics and Functional Morphology Laboratory, CIPER, Faculdade de Motricidade Humana, Universidade de Lisboa, 1499-002 Cruz Quebrada-Dafundo, Portugal; veramps@fmh.ulisboa.pt (V.M.-P.); sarah.mfber@gmail.com (S.B.); apveloso@fmh.ulisboa.pt (A.P.V.); fcarnide@fmh.ulisboa.pt (F.C.); 2 Volkswagen Autoeuropa—Industrial Engineering & Lean Management, Quinta da Marquesa, 2954-024 Palmela, Portugal; carlos.fujao@volkswagen.pt

**Keywords:** short-term musculoskeletal pain, biomechanical factors, posture, force, exposure

## Abstract

To determine the short-term associations between biomechanical risk factors and musculoskeletal symptoms in the upper limbs and low back in an automotive company, a longitudinal study with a follow-up of 4 days was conducted in a sample of 228 workers of the assembly and paint areas. Data were analyzed using generalized estimating equations, calculating the crude and adjusted model for age, sex, seniority, and intensity of pain at baseline. The interactions found were the same for both models. Workers were divided in low-risk and high-risk group for posture, force, exposure, percentage of cycle time with the arm at/above shoulder level, and with the trunk flexed or/and strongly flexed. The predictive factors showed by time × group effect were found between pain intensity on the left shoulder for posture (β = 0.221, *p* < 0.001), percentage of time with the trunk flexed (β = 0.136, *p* = 0.030) and overall exposure (β = 0.140, *p* = 0.013). A time × group interactions were observed, namely between neck pain and posture (β = 0.218, *p* = 0.005) and right wrist and force (β = 0.107, *p* = 0.044). Workers in the high-risk group were more prone to report unfavorable effects on their self-reported musculoskeletal pain, across a workweek when exposed to specific risk factor, being posture important to neck, right wrist and left shoulder pain.

## 1. Introduction

Musculoskeletal disorders (MSDs) and symptoms (MSSs) are the most common work-related health problem in the European Union, impacting workers and employers across all economic sectors and occupations. Besides, MSDs have a high economic and social burden, affecting not only companies and businesses but also society’s health care systems [1].

Data from 31,612 workers reported in the 2015 sixth wave of the European Working Condition Survey showed that three out of five workers in the European Union-28 had MSDs [1]. The most common complaints were back (43%) the upper limbs (41%) pain. Moreover, when questioned about the pain during the previous 12-months, 47% of the plant and machine operators and assemblers reported musculoskeletal pain (MSPs) in the shoulders, neck, and/or upper limbs, whereas 55% reported back pain [1]. Thus, this is one of the occupations with the highest prevalence of reported MS complaints [1]. Simple tasks such as tightening, picking up, and material handling, performed in the automotive production line have been suggested as the culprit behind the high incidence of MSDs [2]. These types of operations have highly repetitive tasks, forceful exertion, and awkward postures, among other known biomechanical risk factors [3,4,5]. Furthermore, short work cycles and insufficient recovery time related to the assembly line have often cumulative effects on mechanical load in the exposure during the work shift [5,6,7].

Among the most subjective symptoms of MSDs are sensations of constant muscle fatigue and stiffness accompanied by radiating pain [8]. Despite the fact that MSDs are one of the most common health problems in the automotive industry [9,10], where heterogeneous work tasks may be found [11], short-term pain trajectory (e.g., one week) has received limited attention in the workplace. Most of the literature addressing the importance of perceived symptoms has focused on the cross-sectional [5,12] and long-term longitudinal [11,13,14,15] associations between physical and, psychosocial factors, and MSSs with no studies addressing the short-term associations in the automotive industry. Understanding how early MSSs, before or after a work shift, evolve throughout a workweek, while exposed to different biomechanical risk factors, may provide valuable insight on the progression of symptoms and prevention of MSDs, such as which time of the day may be more sensitive to detect differences in pain reporting [6,16]. In fact, when looking at exposure-response models, mainly on the short-term effects, the repercussions of the external exposure (i.e., biomechanical risk factors such as posture, force, etc.) on internal exposure (acute responses at system, tissue, cellular, and molecular level) during the working day and some hours after, may have serious medium to long-term implications on workers’ health if not followed by a proper recovery [17]. This issue is of utter importance, since these short-term effects may lead to more permanent symptoms and/or clinical disorders, most of the time accompanied by a decrease in work capacity and a negative impact in the productivity [18].

Therefore, this study aims to determine the prospective associations between biomechanical risk factors and MSPs in the upper limbs and low back in a production line of an automotive company throughout a workweek.

## 2. Materials and Methods

### 2.1. Study Design

This research has a prospective study design, which was conducted between June and July 2019 among a production line of a large automotive company.

### 2.2. Participants

A total of 302 workers (α = 5%, β = 0.80, d = 0.5, 20% of MSSs prevalence in the automotive industry, and a 15% drop-out) [19] divided into 16 randomly selected teams from the assembly and paint areas, were invited to participate in this study. This sample was initially selected from a broader project aiming to develop a mathematical formulation to generate job rotation plans to teams in the production line of an automotive industry.

The study involved one week of work, which started after two days off, followed by 5 consecutive days of work. The eligibility criteria included having a contract with the company, being allocated to assembly and paint areas, having at least 3 months of seniority, not have any medical restrictions to perform the job assessed by the plant medical doctor, and not being a temporary worker. Workers and management in the company were informed at the organizational level first. The week before starting the data collection, the researcher met with all the workers from each team to explain the study aim, protocol, and provide detailed information on how to proceed during the data collection period. All participants gave their written informed consent before their participation in the study.

### 2.3. Self-Reported Musculoskeletal Pain

During the workweek (4 consecutive days), workers were asked to report their daily pain intensity in 10 body regions (neck, right and left shoulder, right and left elbow, right and left wrist, right and left hand/finger, and low back) using a numeric rating scale [20]. In this scale, workers reported a number between 0 and 10 that fitted their MSPs intensity, where 0 represents “no pain” and 10 “the worst pain” [20]. On the first day, the researcher individually handled the questionnaires to workers and explained how to fill them out throughout the week. Every day the workers reported their pain intensity immediately before and after the shift. The pain intensity reported at the beginning and at the end of the shift was used in the analysis.

The job rotation plan and the workstations assigned for each worker, during the data collection week, were provided by each Team Leader. The job rotation plan of each worker was collected to provide information about their individual daily and weekly exposure from each workstation.

### 2.4. Biomechanical Risk Factors

The biomechanical risk factors were assessed using the European Worksheet Method (EAWS), by certified ergonomists working within the company. This method is often used and validated in the automotive industry [21]. The theoretical model that supports this method overcome the traditional concept of limiting values of NIOSH (recommended weight lifting) [22] and in the ISO 11226, ISO 11228-1, ISO 11228-2, and ISO 11228.

The EAWS method results in a traffic light scheme point to classify the exposure severity level of each workstation evaluated. EAWS is divided in four sections for the evaluation of (1) working postures and movements with low additional physical efforts; (2) action forces of the whole body or hand-finger system; (3) manual material handling (>3 kg); (4) repetitive loads of the upper limbs.

#### 2.4.1. Posture

In the first section, static working postures and high frequent movements were estimated. Working postures for standing, sitting, bending, kneeling, crouching, lying, and climbing were rated. Asymmetric postures for the trunk, such as trunk rotation, lateral bending, and far reach, were also evaluated. For this section the longer the time spent in unfavorable conditions, the higher the score for this risk factor.

##### Posture—Percentage of Cycle Time

Within the partial scores for posture, the variables percentage of cycle time with the arm at/above shoulder level (%CT shoulder), and percentage of cycle time with the trunk bent or strongly bent (%CT bent) were defined as the percentage of time that each worker is exposed to these awkward postures during the cycle time of that workstation.

#### 2.4.2. Force

Whole body and hand-finger action forces above 30 to 40 N, respectively, were considered in the second section of the EAWS method. A total score for force was derived by multiplying the intensity and the duration (static)/frequency (dynamic) of force exertions. Finally, the variable exposure represents the total score for a specific workstation and the variables posture and force were defined by the partial scores for each of these risk factors.

### 2.5. Demographic Data

Demographic data concerning age, sex, and seniority for all workers was collected by documental search from Human Resources Department and was provided by the company before the assessments.

### 2.6. Statistical Analysis

Given the high drop-out rate on the 5th day of assessments, only the first 4 days were considered in the analyses. A descriptive analysis was carried out to present sample baseline characteristics and mean scores for MSPs over the 4 days follow-up period. Mean scores and standard deviation were calculated for the whole population and for the sub-groups that were defined according to the different risk factors: posture, force, %CT shoulder, and %CT bent. These sub-groups were established based on the tertiles of the EAWS results. The low-risk sub-group included the first 2 tertiles and the high-risk group, the upper tertile. Thus, the cut-offs to be allocated in each of the high-risk groups were: having a total exposure score above 33.63; a posture score above 20.39; for force risk factor a score above 8.21 points; for the risk factor %CT shoulder a score above 10.18, and the %CT bent risk factor a score above 10.59.

Comparisons between groups (low risk and high-risk groups for each of the EAWS variables) at baseline were performed using the parametric independent sample *t*-tests for those normally distributed outcomes (i.e., age, seniority) and the non-parametric Mann–Whitney test in the absence of normality distribution on the variables (i.e., self-reported pain).

Generalized estimating equations (GEE) were used to analyze the between-group and within-group changes for MSP and the least significant differences were used for post hoc test [23,24]. Unadjusted models were performed as well as models adjusted for potential confounding factors including age, seniority, gender, and baseline symptoms if differences between groups at baseline were observed. A linear distribution for the response was assumed and an autoregressive correlation matrix was set to the data [23,24].

Statistical analysis was performed using IBM SPSS Statistics version 25.0 (SPSS Inc., an IBM company, Chicago, IL, USA). For all tests, statistical significance was set at *p* < 0.05.

## 3. Results

### 3.1. Sample Characteristics and Exposure

All the 302 workers, who were invited to participate in the study, filled the baseline questionnaire. However, the final sample included 228 workers, since 74 had to be excluded given the lack of ID in the follow-up questionnaires. The decision to remove the fifth day was justified by the dropout rate on the final day of the workweek.

The baseline characteristics of workers are presented in Table 1. The workers’ mean age was 30.0 ± 7.1 years, the seniority was 2.0 ± 3.8 years and 39.5% were females. In the total exposure and posture groups, statistically significant differences were found in seniority between groups. There were no statistical differences in the workers’ mean age and between genders across the exposure groups.

### 3.2. Musculoskeletal Symptoms Tendency according to Work Exposure

**Figure 1:**
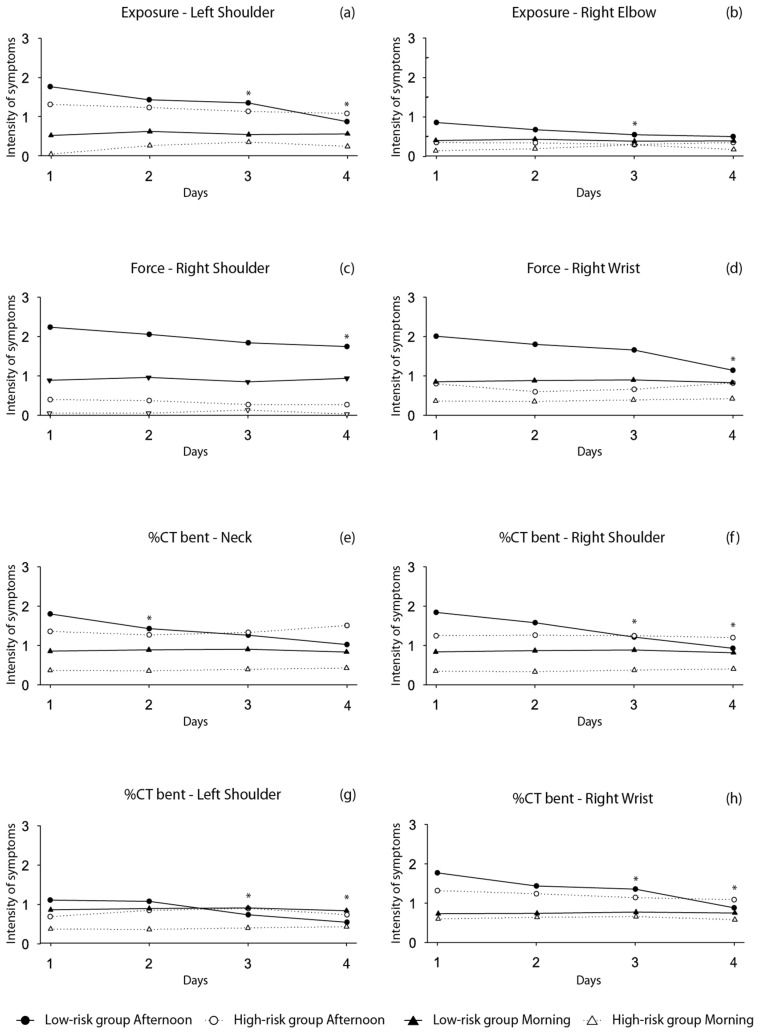
Trajectory of MSP reported at the beginning and the end of the shift during the 4 period of data collection. (**a**) Within-group changes are shown for exposure and left shoulder; (**b**) within-group changes are shown for exposure and right elbow; (**c**) within-group changes are shown for force and right shoulder; (**d**) within-group changes are shown for force and right wrist; (**e**) within-group changes are shown for %CT bent and neck; (**f**) within-group changes are shown for %CT bent and right shoulder; (**g**) within-group changes are shown for %CT bent and left shoulder; (**h**) within-group changes are shown for %CT bent and right wrist. * Within-group changes for the afternoon group significant at *p* < 0.05.

**Figure 2:**
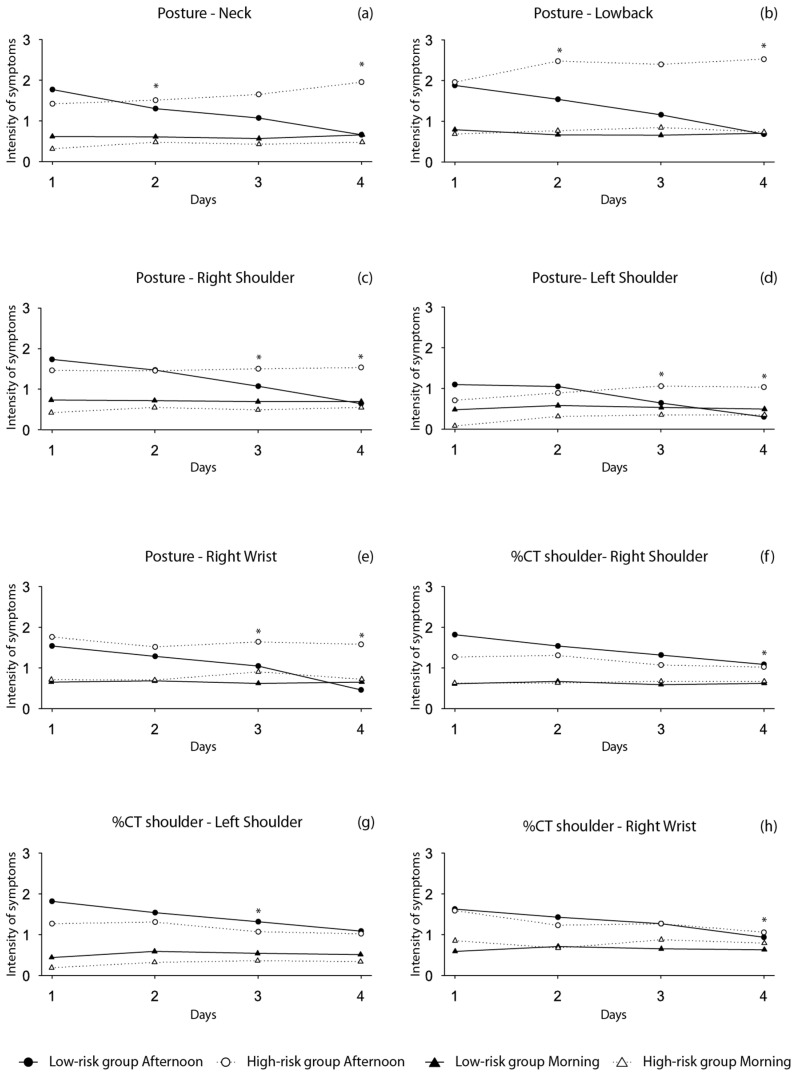
Trajectory of MSP reported at the beginning and at the end of the shift during the 4 period of data collection. (**a**) Within-group changes are shown for posture and neck; (**b**) within-group changes are shown for posture and lower back; (**c**) within-group changes are shown for posture and right shoulder; (**d**) within-group changes are shown for posture and left shoulder; (**e**) within-group changes are shown for posture and right wrist; (**f**) within-group changes are shown for %CT. Shoulder and right shoulder; (**g**) within-group changes are shown for %CT shoulder and left shoulder; (**h**) within-group changes are shown for %CT shoulder and right wrist. * Within-group changes for the afternoon group significant at *p* < 0.05.

Figure 1 and Figure 2 depict the information of the within-group changes throughout the workweek on the pain reported in different body segments assessed at the beginning and the end of the shift, in workers categorized in the high vs low-risk group according to exposure, force, posture, %CT shoulder and %CT bent risk factors. We found a within-group changes with a negative trend for the pain reported on both shoulders and right wrist in those categorized as the low-risk group in all the risk factors (*p* < 0.05). Similarly, we observed a negative trend throughout the week for neck pain symptoms reported by the low-risk group in what concerns posture and %CT bent risk factors, and for low back pain in the posture low-risk group. Conversely, we observed no within-group changes in the MSP reported at the beginning of the shift throughout the week.

### 3.3. Predictive Models of Musculoskelatal Pain

Table 2 summarizes the results for the within and between-group interaction effects with each biomechanical risk group, adjusted for age, gender, seniority, and baseline values whenever differences were found between groups for baseline measurements. Following adjustments, most of the between-group effects were found at the end of the shift. As a result, those the predictive factors are allocated to the high-risk group for the exposure (β = 0.140, *p* = 0.013), posture (β = 0.221, *p* < 0.001), and %CT bent (β = 0.136, *p* = 0.030) had a significant interaction for pain symptoms reported in the left shoulder when compared to those in the low-risk group. For the pain reported in the neck and right wrist region, we can conclude that the predictive factors with significant interaction effect in the high-risk group were posture (β = 0.218, *p* = 0.005) and force risk factors (β = 0.107, *p* = 0.044), respectively. Finally, at the beginning of the shift, the left shoulder region also had an interaction effect for posture in those in the high-risk group (β = 0.053, *p* = 0.008), when compared to those in the low-risk group.

## 4. Discussion

This study aimed to determine the prospective associations between biomechanical risk factors and MSPs in the upper limbs and low back in a production line of an automotive company throughout a workweek.

To our knowledge, this is the first study to analyses the prospective short-term associations between biomechanical risk factors and MSP in the upper limbs and low back, in a production line of an automotive company during a workweek. The main findings were that during this period the intensity of self-reported MSP was less favorable in the high-risk group, for selected biomechanical risk factors, such as overall exposure, force, posture, and %CT bent, specifically on neck, shoulder, and wrist segments, when compared with the low-risk group. These associations were more pronounced after the shift when compared to the beginning of the shift. These results suggest that workers in the high-risk groups of these specific risk factors may be more susceptible to have increased MSP. Thus, if continuous exposure to such conditions is maintained, these workers will have greater odds for future MSDs.

Given the MSDs’ impact in the occupational context, more specifically in the automotive industry, it is paramount to understand which specific risk factors increase the incidence of MSDs and how to assess and detect early signs and symptoms of this condition. Our results add upon the current literature by showing that one week of work can alter the self-reported MSP of workers in the automotive industry depending on the exposure to a given risk factor and the body segment analyzed. For instance, and considering the posture risk factor, workers who were categorized in the high-risk group had higher MSPs scores for both neck and left shoulder body regions. Moreover, the shoulder region was also identified as a specific region of interest, since a time × group interaction was also found for exposure, and the %CT bent risk factors, favoring the low-risk group. Likewise, we also observed time × group interaction in the force risk factor for the right wrist region. Given that posture has been identified as an established risk factor for MSDs [10,25,26,27] and since it is composed by %CT shoulder and %CT bent, there was either a within-group changes alone or time × group interaction for the left shoulder, special attention should be given to this risk factor, on the short-term management of workers exposure. The literature on the MSDs incidence and the connection between self-reported pain for the neck and posture risk factor [28,29], as well as, wrist pain and force risk factor has also been established [30], and hence should also be monitored. Beyond the between-group effects, within group changes were also found between different body regions and all risk factors, reinforcing the notion that those in the low-risk group may also benefit from the decreased intensity of self-reported pain over the working week. These changes can be observed across all risk factors, being the right wrist, right shoulder, and left shoulder the most affected regions. Even though it was absent from the between group changes, the low back region had a time-effect for the low-risk group in the posture risk factor. This region has been previously identified as one of the body segments with a high prevalence for MSSs [31], in which posture is considered a risk factor [10]. In fact, when considering other industries, frequent occupational lifting has been associated to short-term increase in reported low back pain. Our study found similar results to those of Andersen at al. [32] where the increase in pain intensity was of small magnitude during the study period. Nonetheless, our study did not assess occupational lifting, but unfavorable postures adopted by workers during the workday may be the underlying cause of low back pain [2].

Our results are in accordance with previous studies, some with cross-sectional [5], others with long-term prospective designs [6,13] showing that disorders in the upper limbs such as shoulders, and wrists increased markedly with overall exposure scores, composed by biomechanical risk factors such as awkward postures and forceful exertions. Regarding neck self-reported pain, Da Costa et al. [13], in a systematic review of prospective studies, also provided evidence on the connection between awkward postures and increased symptomatology in this body region, across several industries and workplaces. However, none of these studies accessed self-reported pain in the short-term (i.e., such as during a workweek), which might provide valuable insight into early symptoms, since a shorter duration of shoulder MSP, among others, is a predictor of greater improvement in disability [33]. Therefore, assessing MSP during a shorter period could be a way to prevent or help improve the outcomes of an injury, thus affecting the incidence of long-term MSDs.

In this study, even though the average intensity of the self-reported MSP were scored as mild (1–3) [34], it is still noteworthy that the interaction observed on the exposure to these specific risk factors might help, through a cumulative manner, on the management of long-term risk for MSD [33]. For instance, for the left shoulder to be in the high-risk group for the overall exposure, posture and %CT bent was associated with increased self-reported pain intensity, during a 4-day work period, which in the long-term may accrue the pain’s intensity to a cut-off value closer to three (scale: 1–10). In fact, this value was identified in the literature as a criterion in the diagnosis of rotator cuff tendonitis [35]. On this topic, the work developed by the team leaders at the production line on managing the rotation plans may prevent or aggravate the exposure to these risk factors. The team leaders’ rotation plans are made empirically, and without considering the evaluation carried out by the validated evaluation method EAWS [21]. Nonetheless, they are trained to actively pursue weekly changes in diversity and variability in overall exposure and thus, mitigate the effects of the cumulative exposure to the biomechanical risk factors, reducing the incidence of the MSPs. Another important factor, concerns the initial condition of all workers, regardless of their previous week, where they start their workweek following a resting period of 2 days. We can speculate that both the rotation plan and the 2-day rest period may impact the pain intensity reported by the workers and reset their perceived symptomatology at the beginning of each week. However, even if there is residual pain from the workweek prior to the assessment, we adjusted all models when baseline differences were observed for self-reported MSP. Therefore, the time × group interactions between the high and low risk group for each of the body segments were irrespective of the worker’s baseline values.

Another finding from this investigation concerns the results obtained at the end and the beginning of the shift, throughout the workweek. Most of the associations found for the within-group changes and time × group interactions were observed at the end of the shift, which could be explained by the fact that workers had already undergone their shift, thus were already exposed to all of the risk factors. Interestingly, there was no within-group changes for the MSP reported at the beginning of the shift throughout the week, suggesting that workers always started on average with the same intensity of self-reported pain. Therefore, self-reported pain at the end of the shift may provide more valuable information, especially if looking at the short-term cumulative effects for exposure. To our knowledge, most of the literature does not report the time of the working day when the pain was reported. In fact, one study in a seafood processing factory indicated that the data collected was performed after the shift, also found that 80% of their workers reported symptoms after the shift on upper and lower extremities, neck, and shoulders [16].

This study is not without limitations. For instance, we did not control the models for the risk factors that workers were exposed in the week prior to data collection, and other important determinants, such as physical activity level and the handedness of the participants. However, when baseline differences between groups were observed for MS intensity pain, we adjusted the models for baseline values in each of the groups [36]. Regardless of the initial briefing on how to fill the questionnaires, self-reporting data on MSP can always be biased depending on workers’ mood and on a higher frequency of data collection. Despite the dropout being higher than expected (~30%), the 228 subjects included in the final sample still have a high variability of exposure to the risk factors, given the tasks performed in the production line. Moreover, the results obtained in the intention to treat analysis (data not shown) did not differ from those presented in this study.

One of the methodological strengths of our study is the short-term longitudinal approach, which might provide valuable insight into how MSP may be related to biomechanical risk factors. Additionally, we provide information in several body segments, while also accounting for different biomechanical risk factors in both the beginning and end of the shift using a significant sample size.

## 5. Conclusions

In conclusion, this work suggests that workers in the high-risk groups to biomechanical risk factors such as posture, force, and the overall exposure had unfavorable effects on their self-reported MSSs throughout a workweek. More specifically, the risk factor posture seems to have an increased contribution to the MSSs in the neck and left shoulder regions. Therefore, alternating exposure to such risk factors may be of relevance to the short-term period to possibly prevent or help improve the MSSs, thus affecting the incidence of long-term MSDs in the automotive industry.

## Figures and Tables

**Table 1:** Sample characteristics, according to each of the risk factors and low-risk and high-risk groups.

	Exposure		Posture		Force		%CT Shoulder		%CT Bent		Total Sample (*n* = 228)
	Low Risk (*n* = 152)	High Risk (*n* = 76)	Low Risk (*n* = 152)	High Risk (*n* = 76)	Low Risk (*n* = 152)	High Risk (*n* = 76)	Low Risk (*n* = 152)	High Risk (*n* = 76)	Low Risk (*n* = 152)	High Risk (*n* = 76)	
Age (years)	30.3 ± 7.4	29.7 ± 6.5	30.4 ± 7.3	29.4 ± 6.7	30.1 ± 6.8	29.8 ± 7.6	29.7 ± 6.9	30.7 ± 7.4	30.2 ± 7.2	29.6 ± 6.9	30.0 ± 7.1
Seniority (years)	2.3 ± 4.5	1.2 ± 1.6 *	2.9 ± 4.4	1.3 ± 2.2 *	2.0 ± 3.9	1.8 ± 3.6	1.9 ± 3.9	2.0 ± 3.7	2.1 ± 4.0	1.6 ± 3.4	2.0 ± 3.8
Gender (% female)	39.1	40.8	39.5	39.5	38.4	42.1	44.1	30.3	36.8	44.7	39.5

* Significance *p* < 0.05; %CT shoulder—percentage of cycle time with the arm in extreme posture (at/above shoulder level); %CT bent—percentage of cycle time with the trunk bent or strongly bent.

**Table 2:** Within and between-group changes in musculoskeletal symptoms in different body regions for different biomechanical risk factors, after a workweek. Betas (β) are presented as unstandardized coefficients adjusted. The model is adjusted for gender, age, seniority, and baseline whenever differences were found between the low-risk and high-risk groups, with the respective 95% confidence intervals.

Pain at the Beginning of the Shift					
	Exposure	Posture	Force	%CT Shoulder	%CT Bent
Body Regions	High Risk * Low Risk β (95%CI)	High Risk * Low Risk β (95%CI)	High Risk * Low Risk β (95%CI)	High Risk * Low Risk β (95%CI)	High Risk * Low Risk β (95%CI)
Neck	0.010 (−0.103–0.123)	0.089 (−0.015–0.193)	−0.049 (−0.156–0.058)	−0.046 (−0.172–0.081)	0.028 (−0.085–0.141)
Low Back	0.020 (−0.112–0.152)	0.124 (−0.001–0.249)	0.007 (−0.106–0.120) ^†^	−0.002 (−0.147–0.143)	0.044 (−0.084–0.173)
Right Shoulder	0.010 (−0.107–0.126)	0.093 (−0.021–0.208)	0.013 (−0.085–0.111) ^†^	−0.047 (−0.178–0.084)	0.014 (−0.107–0.136)
Left Shoulder	0.055 (−0.010–0.119) ^†^	0.053 (0.002–0.104) *^,†^	−0.033 (−0.097–0.030) ^†^	0.035 (−0.035–0.104)	0.054 (−0.021–0.129)
Right Elbow	0.018 (−0.036–0.072)	0.013 (−0.042–0.067)	0.012 (−0.039–0.064)	0.011 (−0.042–0.064)	−0.010 (−0.070–0.050)
Left elbow	0.040 (−0.009–0.089) ^†^	0.026 (−0.014–0.067) ^†^	0.018 (−0.036–0.072)	0.000 (−0.054–0.054)	−0.015 (−0.083–0.054)
Right wrist	0.008 (−0.116–0.106)	0.050 (−0.058–0.159)	0.068 (−0.017–0.153) ^†^	−0.029 (−0.149–0.091)	0.035 (−0.075–0.144)
Left wrist	−0.012 (−0.091–0.068)	0.064 (−0.019–0.147)	−0.009 (−0.087–0.070)	0.006 (−0.081–0.093)	0.017 (−0.068–0.102)
Right hand/fingers	0.005 (−0.107–0.117)	0.038 (−0.075–0.1519	−0.027 (−0.130–0.076)	−0.050 (−0.177–0.076)	0.003 (−0.112–0.118)
Left hand/fingers	0.002 (−0.091–0.094)	0.025 (−0.039–0.089)	−0.019 (−0.103–0.064)	−0.040 (−0.147–0.067)	0.051 (−0.046–0.147)
**Pain at the End of the Shift**					
	**Exposure**	**Posture**	**Force**	**%CT Shoulder**	**%CT Bent**
**Body Regions**	High Risk * **Low Risk β** **(95%CI)**	High Risk * **Low Risk β** **(95%CI)**	High Risk * **Low Risk β** **(95%CI)**	High Risk * **Low Risk β** **(95%CI)**	High Risk * **Low Risk β** **(95%CI)**
Neck	0.002 (−0.150–0.154)	0.218 (0.067–0.368) *	−0.002 (−0.129–0.126) ^†^	0.015 (−0.131–0.162)	0.113 (−0.041–0.267)
Low Back	0.063 (−0.107–0.233)	0.143 (−0.026–0.311)	0.108 (−0.036–0.252) ^†^	−0.008 (−0.180–0.164)	0.054 (−0.111–0.220)
Right Shoulder	−0.027 (−0.169–0.115) ^†^	0.092 (−0.060–0.245)	0.030 (−0.106–0.167) ^†^	0.030 (−0.127–0.188)	0.080 (−0.066–0.227)
Left Shoulder	0.140 (0.030–0.251) *^,†^	0.221 (0.102–0.339) *	0.004 (−0.108–0.117) ^†^	0.075 (−0.049–0.199) ^†^	0.136 (0.013–0.260) *
Right Elbow	0.007 (−0.068–0.082) ^†^	0.055 (−0.042–0.152)	0.015 (−0.057–0.088) ^†^	−0.010 (−0.117–0.098)	0.011 (−0.060–0.081) ^†^
Left elbow	0.031 (−0.040–0.102) ^†^	0.067 (−0.016–0.150)	0.007 (−0.064–0.078) ^†^	−0.008 (−0.092–0.077)	0.016 (−0.071–0.102)
Right wrist	0.005 (−0.131–0.141)	0.020 (−0.114–0.153)	0.107 (0.003–0.211) *^,†^	−0.050 (−0.191–0.090)	0.084 (−0.049–0.218)
Left wrist	0.056 (−0.071–0.183)	0.103 (−0.029–0.235)	0.081 (−0.041–0.203)	0.039 (−0.107–0.186)	0.065 (−0.060–0.191)
Right hand/fingers	−0.084 (−0.299–0.061)	−0.013 (−0.157–0.130)	−0.001 (−0.126–0.125) ^†^	−0.046 (−0.199–0.107)	−0.094 (−0.239–0.052)
Left hand/finger	−0.062 (−0.175–0.050)	0.051 (−0.070–0.172)	0.047 (−0.057–0.150) ^†^	−0.014 (−0.142–0.114)	0.063 (−0.055–0.180)

* Between-group changes significant at *p* < 0.05; 95%CI—95% confidence interval; ^†^ within-group changes significant at *p* < 0.05; %CT shoulder—the percentage of cycle time with the arm in extreme posture (at/above shoulder level); %CT bent—the percentage of cycle time with the trunk bent or strongly bent.

## Data Availability

The data presented in this study are available from the corresponding author on reasonable request.
